# Novel phenotypic variant in the MYH7 spectrum due to a stop-loss mutation in the C-terminal region: a case report

**DOI:** 10.1186/s12881-017-0463-y

**Published:** 2017-09-19

**Authors:** Zsolt Bánfai, Kinga Hadzsiev, Endre Pál, Katalin Komlósi, Márton Melegh, László Balikó, Béla Melegh

**Affiliations:** 1 Department of Medical Genetics, University of Pécs, Szigeti út 12, Pécs, H-7624 Hungary; 2 Szentágothai Research Centre, University of Pécs, Ifjúság út 20, Pécs, H-7624 Hungary; 3 Neurology Clinic, University of Pécs, Rét u. 2, Pécs, H-7623 Hungary; 4 Department of Neurology, Zala County Hospital, Zrínyi u. 1, Zalaegerszeg, H-8900 Hungary

**Keywords:** Axial muscle atrophy, Myosin storage myopathy, *MYH7*, Stop loss mutation, Case report

## Abstract

**Background:**

Defects of the slow myosin heavy chain isoform coding *MYH7* gene primarily cause skeletal myopathies including Laing Distal Myopathy, Myosin Storage Myopathy and are also responsible for cardiomyopathies. Scapuloperoneal and limb-girdle muscle weakness, congenital fiber type disproportion, multi-minicore disease were also reported in connection of *MYH7*. Pathogeneses of the defects in the head and proximal rod region of the protein are well described. However, the C-terminal mutations of the *MYH7* gene are less known. Moreover, only two articles describe the phenotypic impact of the elongated mature protein product caused by termination signal loss.

**Case presentation:**

Here we present a male patient with an unusual phenotypic variant of early-onset and predominant involvement of neck muscles with muscle biopsy indicating myopathy and sarcoplasmic storage material. Cardiomyopathic involvements could not be observed. Sequencing of *MYH7* gene revealed a stop-loss mutation on the 3-prime end of the rod region, which causes the elongation of the mature protein.

**Conclusions:**

The elongated protein likely disrupts the functions of the sarcomere by multiple functional abnormalities. This elongation could also affect the thick filament degradation leading to protein deposition and accumulation in the sarcomere, resulting in the severe myopathy of certain axial muscles. The phenotypic expression of the detected novel *MYH7* genotype could strengthen and further expand our knowledge about mutations affecting the structure of MyHCI by termination signal loss in the *MYH7* gene.

## Background

Defects of the *MYH7* gene mainly result in myopathic cardiac diseases and skeletal myopathies including distal myopathy and other skeletal muscle abnormalities caused by thick filament accumulation in the sarcomeres. However, according to the reports from recent years, phenotypic characteristics of myopathies arising from *MYH7* gene defects actually have a rather wide spectrum. The high diversity of phenotypic features is based not only on the type of the gene alteration, but the location of the mutation in the *MYH7* gene bears also high importance.

The *MYH7* gene (NM_000257) encodes the slow or beta-cardiac myosin heavy chain (MyHCI). Unlike six of the myosin heavy chain coding genes, which are forming a tightly linked cluster on chromosome 17, *MYH7* can be found on the long arm of chromosome 14 along with *MYH6*. *MYH7* is 22,883 bp long and consists of 40 exons. 38 exons of the gene are involved in the encoding of the 1935 amino acids of MyHCI, and two of the 5-prime exons are untranslated regions. Exons 3–21 are encoding the globular head region including the head and neck, exons 22–40 are responsible for the encoding of the rod region of MyHCI, which consists of the hinge and the light meromyosin chain.

Myosin is the molecular engine of the muscle fiber and converts chemical energy into mechanical work [1]. In a structural level, myosin can be described as a hexamer protein consisting of two myosin heavy chain subunits each attached to two structurally different light chains, of which one is an alkali light chain, while the other is a regulatory light chain subunit [1, 2]. The MyHCI is one of the myosin heavy chain classes and is expressed in the normal human ventricles. MyHCI can also be found in skeletal muscles rich in slow-twitch type I muscle fibers [1]. These skeletal muscles are characterized by slow contraction and mainly responsible for normal posture [3].

MyHCI is in vivo a dimer protein. The globular head region of the myosin molecule is responsible for microfilament motor activity as it has both actin-binding capability and an actin-dependent ATPase activity. The head region attaches to a long rod region, which has an α-helical coiled coil structure. The main function of the rod region is the electric charge based sorting of myosin filaments into thick filaments [4, 5].

Mutations of *MYH7* gene resulting in cardiomyopathic symptoms are caused mainly by heterozygous missense point mutations with a number over 230 [6]. These are connected predominantly to the defects of the head and proximal rod region of the MyHCI molecule.

There are known pathogenic mutations in the proximal rod region of MyHCI which do not cause cardiomyopathy and are responsible for the symptoms associated with Laing Distal myopathy (MPD1; OMIM#160500) [7, 8].

The relatively few known pathogenic mutations of the distal rod region have a different pathogenesis [9]. There are five known mutations in exons 37–39 (p.K1784del, p.L1793P, p.R1845W, p.E1883K and p.H1901L) that are primarily responsible for Myosin Storage Myopathy (MSM; OMIM#608358) [10–14]. Two papers reported mutations involving exon 40 and resulting in termination signal loss and elongation of the protein. These mutations are at the C-terminal light meromyosin region consisting of 29 amino acids and have well known thick filament forming functions [14–16]. The first paper reported that the investigated patient with the c.5807A > G stop-loss mutation was initially diagnosed with congenital fiber type disproportion (CTFD; OMIM #255310) with the predominance of type I fibers, and MSM developed afterwards [17]. The second paper described a patient with distal axial and proximal muscle weakness and distinctive cores in muscle fibers at muscle biopsy. The symptoms were caused by a c.5808G > C mutation [18].

While defects of the head region and proximal rod region of MyHCI protein predominantly result in cardiomyopathic symptoms and Laing Distal Myopathy, mutations of the distal rod region involves primarily MSM and patients do not show the signs of cardiomyopathy [11, 13, 19, 20].

## Case presentation

**Fig. 1:**
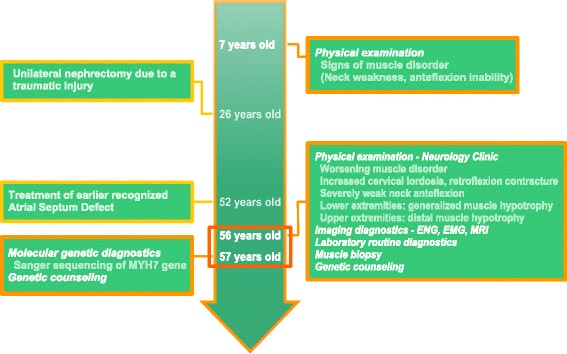
Relevant history of the patient organized into a timeline

A 57 years old male patient was examined at the outpatient department of Neurology Clinic and by our muscle disorder specialist genetic counselors. Relevant history of the patient are summarized on a timeline (Fig. 1). In the medical history he had unilateral nephrectomy due to traumatic injury at the age of 26. Atrial septum defect (ASD) was known since his childhood and it was closed via endovascular route when he was 52 years old.

The family history was unremarkable. In the case of his parents, 3 siblings and his daughter neuromuscular disease was not reported.

His symptoms indicating muscle disorder appeared at age 7. Neck muscle weakness, especially inability of anteflexion was pronounced since the beginning of the disease. In the last few years the neck weakness worsened and he was unable to bend his neck at all. His head tended to fall backwards and he must hold his head with his arms in standing position and walking. Weakness of the extremities was not significant. Recent neurologic examination showed markedly increased cervical lordosis and retroflexion contracture. The neck anteflexion was severely weak (MRC 2/5), the retroflexion was almost normal (5−/5). The facial and the anterior neck muscles were atrophic, bilateral scapular winging was present. Generalized muscle hypotrophy was seen in lower extremities, and distal hypotrophy in upper extremities. The muscle strength was generally decreased (MRC 4–4+/5) without characteristic distribution, deep tendon reflexes were absent in all extremities. There was no sensory deficit, dysphagia or involvement of ocular, cardiac and respiratory muscles.

**Fig. 2:**
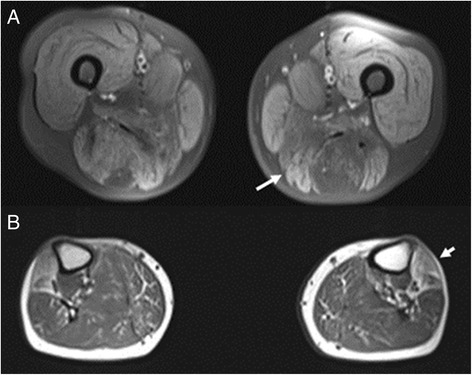
Axial MRI of the thigh (**a**) and the calf (**b**). **a**. T1 fatsat image shows fatty degeneration of muscles in the posterior compartment (dark, arrow). **b**. T1 image shows degeneration of anterior tibial muscles (arrow) and fatty infiltration of medial gastrocnemius muscles

Imaging studies showed atlanto-axial instability without spinal cord lesion. Brain MRI was normal. Electromyography (EMG) of proximal muscles resulted in small, myopathic unit potentials and increased polyphasy. Electroneuronography (ENG) was negative. Laboratory tests were normal including creatine kinase (CK). MRI of thigh muscles showed degenerative changes in the posterior compartment and adductor magnus on T1-weighted images. In the calf level anterior (extensor) muscles showed degenerative changes and the medial gastrocnemius muscle showed hyperintensity on STIR study resembling edema and fatty infiltration on T1 (Fig. 2). The pronounced degeneration of anterior tibial muscles observed on MRI showed the main characteristic of *MYH7*-related myopathies [18].

**Fig. 3:**
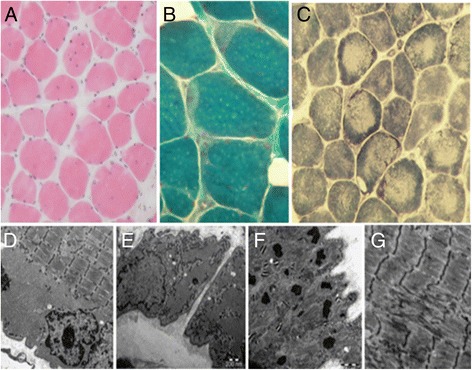
Muscle biopsy results. Myopathic changes: increased fiber-size variation, internal nuclei, endomysial fibrosis (**a**). Subsarcolemmal storage material staining bright on HE (**a**) and with modified Gomori (**b**), dark on NADH (**c**). The storage material beneath the sarcolemma is clearly visible on the electron microscopic (EM) image (**d**). An atrophic fiber is almost completely filled with granular storage material (**e**). Structural alterations of fibers: dysorganized sarcomers and abnormal electrondense bodies (**f**), „core-like” appearance of fibers with NADH (**c**) and core structures on EM (**g**). Original magnifications: A: 80×, B: 160×, C: 80×, D: 10.000×, E: 7.500×, F: 20.000×, G: 15.000×

Biopsy of deltoid muscle showed round, markedly atrophic muscle fibers, increased ratio of internal nuclei and proliferation of connective tissue corresponding to myopathy. In certain muscle fibers subsarcolemmal cytoplasmic body was recognized with eosinophylic appearance on HE, blue staining with modified Gomori and increased enzyme activity with nicotinamide adenine dinucleotide dehydrogenase (NADH) enzyme histochemistry. Few cytochrome-c oxidase (COX) negative fibers were present. Central loss of enzyme activity was seen in several fibers with all oxidative enzyme reactions. Immunohistochemistry for proteins involved in muscular dystrophies was unremarkable. Electron microscopy showed core structures in the central part of some fibers and moderate accumulation of mitochondria in the interfibrillar space. Huge amount of granular sarcoplasmic material was accumulated in the periphery of several fibers (Fig. 3.). This histological pattern suggested congenital myopathy with sarcoplasmic storage material.

**Fig. 4:**
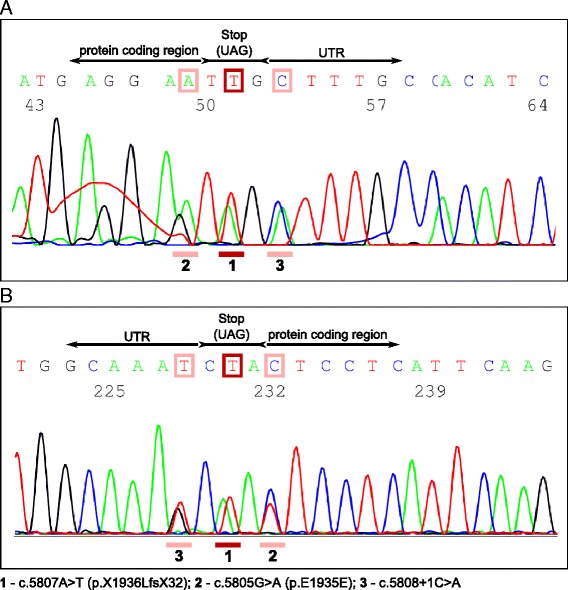
Electropherogram of the involved sequence fragment of *MYH7.* The result of forward sequencing (**a**). The result of reverse sequencing (**b**). The detected mutation and the two variations c.5805G > A, c.5807A > T and c.5808 + 1C > A are highlighted with rectangles in the nucleotide sequence and corresponding peaks are also underlined

Diagnostic Sanger sequencing of the *MYH7* gene revealed a heterozygous adenine-timine base transversion, which involves the next to last base in the coding region of exon 40 and causes the elongation of the protein sequence by 31 amino acids due to the loss of the termination codon, resulting in a leucine coding triplet (c.5807A > T; p.X1936LfsX32). Although no functional studies were performed to investigate the mutation, it was tested applying various prediction software. Mutation Assessor and MutationTaster confirmed its termination signal loss effect [21–23]. MutationTaster predicted also the probable alteration of splicing, the elongation of the gene product and the failure of the coiled coil forming process in the rod region of MyHCI. Analyzing further the probable splice site alteration effect, we applied the Human Splicing Finder v3.0 (HSFv3.0) software, which predicted the formation of an exonic splicing silencer site [24]. The Paircoil2 software was used to further investigate the predicted defect of coiled coil forming function of the rod region [25]. According to Paircoil2, the probability of coiled coil formation was less than 10^−5^. The mutation was evaluated also with the Combined Annotation Dependent Depletion (CADD) tool [26]. CADD predicted the mutation to be deleterious with a significant C-score, which was 21.3. Besides this pathogenic mutation, two additional heterozygous single nucleotide variations in exon 40 were also detected (Fig. 4). One of them was a synonymous variant (c.5805G > A; p. E1935E), the other was a 3-prime UTR variant following the coding region of exon 40 (c.5808 + 1C > A). None of the further detected two variants were reported or could be found in genetic databases dbSNP [27], ClinVar [28], in the Human Gene Mutation Database (HGMD) [29] or in the Leiden Open Variation Database (LOVD) [30]. Therefore we applied the HSFv3.0 and NetGene2 software in order to test whether the variants could be responsible for probable splice site alteration [24, 31, 32]. Results showed that these variants are likely not affecting the splicing process. The two variations were also investigated using CADD. According to CADD results, the synonymous variant was likely neutral. It showed also the probable deleteriousness of the 3-prime UTR variant. Although this variation, found in the untranslated region, is actually involved in the encoding of the elongated gene product caused by the detected stop-loss mutation.

This case report was prepared according to the CARE guidelines.

## Discussion and conclusions

The two earlier referred studies, introducing also novel stop-loss mutations in exon 40 of *MYH7* (Table 1)*,* reported similar phenotypes regarding the absence of cardiac or respiratory system involvement, myopathic EMG and the significance of proximal muscle weakness. The phenotype of c.5807A > G mutation was similar to our case regarding the predominance of proximal skeletal myopathy. Subsarcolemmal storage material was also present. The patient was initially diagnosed with CFTD, which suggests that muscle disorder evolved to MSM later in more advanced stages of the disease. The phenotype of the second reported stop-loss mutation (c.5808G > C) showed a less predominant involvement of proximal muscle weakness by more significant involvement of distal muscles. Muscle biopsy revealed cores in muscle fibers in contrast to the first study and to our case, which both revealed eosinophilic sarcoplasmic storage material during biopsy. Particular characteristics of our case were the early-onset (childhood) myopathy of neck muscles with a very slow progression resulting a severe myopathic phenotype with the predominant myopathy of neck muscles at older age. The early onset and slow progression of the disease along with the observed muscle pathology corresponds to MSM.

**Table 1:** Clinical and histopathological features of the two reported stop-loss mutations and our case

No.	Mutation	Age	Sex	Onset	Family history	Predominantly affected muscles	Cardiac involvement	Respiratory involvement	EMG	Muscle biopsy
1	c.5807 A > G p.X1936Trp	62	M	childhood (as CFTD)	yes	proximal	no	no	myopathic	hyaline bodies
2	c.5808 G > C p.X1936Tyr	39	F	adult	no	distal axial (and proximal)	no	no	myopathic	cores
3^a^	c.5807 A > T p.X1936Leu	57	M	childhood neck weakness	no	proximal, more severe neck weakness	no	no	myopathic	hyaline bodies

^a^our patient, reported in this case report

Pathogenesis of myosin storage myopathy can manifest in many ways from thermodynamic and functional abnormalities of the protein to the disruption of the whole myosin filament forming process. Pathogenicity of this mutation is supported by the fact that elongation of the protein inhibits the forming of the α-helix structure of the rod region. Alternative splicing mechanism in the case of *MYH11* illustrates that merely the length of the protein can affect its flexibility and mechanical properties. Alternative splicing of *MYH11* enables the formation of two protein isoforms. The length of the rod region of the two isoforms differs in 34 base pairs resulting in highly different flexibility properties. These isoforms possess also different functions [33]. The elongated region of MyHCI contains three proline molecules. The acquired proline content disrupts the coiled coil structure formation of the elongated protein, by drastically decreasing its flexibility and also prevents the formation of thick filaments in the sarcomere [34]. Increased proline content might affect the degradation of thick filaments causing protein deposition and accumulation in the sarcomere [35]. A recent study (referred also earlier in the Background section) presented two heterozygote mutations in the proximal rod region (c.4309G > C and c.4309G > C) resulting in proline substitution [7]. These mutations were involved in progressive distal myopathic phenotypes very similar to our case regarding especially the severe myopathy of the neck, but without subsarcolemmal thick filament aggregation. The study pointed out the fact that proline substitution causing mutations in the rod region affect the intrinsic ability of the beta-myosin molecule to establish the coiled coil structure and prevent also the correct filament formation. This study proposed also an explanation to the absence of the sarcoplasmic storage material. The wild type myosin (encoded by the normal allele) likely facilitates the incorporation of the defective MyHCI form into the sarcomere preventing its aggregation. The examples of these two proline substituting *MYH7* mutations show us, that one proline substitution in the rod region does not cause necessarily myopathic symptoms by facilitating protein deposition in the sarcomere. However, in contrast to these mutations, the elongated region caused by the stop-loss mutation incorporates three additional proline molecules, which might have more severe effect to the MyHCI protein structure. Moreover, significant change in protein length and acquired proline content together might be responsible for storage material accumulation in the sarcomeres, shown by the example of c.5807A > G mutation and our present case.

Examining of *MYH7* mutations and the corresponding phenotypic spectrum allows us to better understand the nature of congenital myopathic disorders, helps in the classification of clinical patterns and in the diagnosis of currently unidentified cases. C-terminal mutations of the rod region of MyHCI might have significant role in myopathies caused by autosomal dominant myosin alterations, therefore bearing high importance in differential diagnostics. Number of currently known stop-loss mutations in *MYH7* are few, their impact on the structure of MyHCI is rather unique and do not show a uniform phenotype. This case could expand our knowledge about the phenotypic spectrum of MyHCI mutations regarding especially the possible phenotypic effects of an elongated slow myosin heavy chain.

## Abbreviations

ASD: Atrial septum defect
ATPase: Adenosinetriphosphatase
CADD: Combined Annotation Dependent Depletion
CFTD: Congenital Fiber Type Disproportion
CK: creatine kinase
COX: cytochrome-c oxidase
EMG: Electromyography
ENG: Electroneuronography
HE: Haematoxilyn and Eosin (tissue staining technique)
HGMD: Human Gene Mutation Database
HSFv3.0: Human Splicing Finder v3.0
LOVD: Leiden Open Variation Database
MPD1: Laing Distal Myopathy
MRC: Medical Research Council
MRI: Magnetic resonance imaging
MSM: Myosin Storage Myopathy
MYH11: myosin heavy chain 11, smooth muscle
MYH6: myosin heavy chain 6, cardiac muscle, alpha
MYH7: myosin heavy chain 7, cardiac muscle, beta
MyHCI: slow/beta-cardiac myosin heavy chain
NADH: nicotinamide adenine dinucleotide dehydrogenase
STIR: Short Tau Inversion Recovery (Magnetic resonance imaging)
