# Correction to: Novel phenotypic variant in the MYH7 spectrum due to a stop-loss mutation in the C-terminal region: a case report

**DOI:** 10.1186/s12881-017-0510-8

**Published:** 2017-12-16

**Authors:** Zsolt Bánfai, Kinga Hadzsiev, Endre Pál, Katalin Komlósi, Márton Melegh, László Balikó, Béla Melegh

**Affiliations:** 1 Department of Medical Genetics, University of Pécs, Szigeti út 12, Pécs, H-7624 Hungary; 2 Szentágothai Research Centre, University of Pécs, Ifjúság út 20, Pécs, H-7624 Hungary; 3 Neurology Clinic, University of Pécs, Rét u. 2, Pécs, H-7623 Hungary; 4 Department of Neurology, Zala County Hospital, Zrínyi u. 1, Zalaegerszeg, H-8900 Hungary

## Correction

Following publication of the original article [1], the authors requested a correction to the details of one of the co-authors. Professor Béla Melegh had been incorrectly marked by the typesetters with the ^ sign as “deceased” instead of Márton Melegh.

The original article has been updated.
